# Dr. John Archibald McDonnell

**Published:** 1914-01

**Authors:** 


Dr. John Archibald McDonnell, Buffalo, 1875, Prof. of Surgery in the Bennett Eclectic College of Medicine of Chicago, died at his home in that city, Sept. 19, aged 66.

